# Innate Immune Evasion Strategies by Human Immunodeficiency Virus Type 1

**DOI:** 10.1155/2013/954806

**Published:** 2013-08-12

**Authors:** Debjani Guha, Velpandi Ayyavoo

**Affiliations:** Department of Infectious Diseases and Microbiology, Graduate School of Public Health, University of Pittsburgh, 130 Desoto Street, Pittsburgh, PA 15261, USA

## Abstract

Host immune components play both beneficial and pathogenic roles in human immunodeficiency virus type 1 (HIV-1) infection. During the initial stage of viral infection, a complex network of innate immune factors are activated. For instance, the immune cells express a number of inflammatory proteins including cytokines, chemokines, and antiviral restriction factors. These factors, specifically, interferons (IFNs) play a crucial role in antiviral defense system by modulating the downstream signaling events, by inducing maturation of dendritic cells (DCs), and by activation of macrophages, natural killer (NK) cells, and B and T cells. However, HIV-1 has evolved to utilize a number of strategies to overcome the antiviral effects of the host innate immune system. This review discusses the pathways and strategies utilized by HIV-1 to establish latent and persistent infection by defeating host's innate defense system.

## 1. Introduction

During the early phase of infection, hosts mount innate immune response that comprises defense mechanisms to protect the hosts from invading pathogens in an antigen independent manner. This immune response is the first and a rapid response launched against a variety of microorganisms. The innate immune system can distinguish between self and foreign proteins and responds accordingly. This nonspecific immune response is activated primarily by the structural motifs of invading pathogens. The major cell types that play key roles in innate immune response against invading pathogens include macrophages, dendritic cells, neutrophils, natural killer cells, mast cells, eosinophils, and basophils. Most of the innate effector cells produce inflammatory factors that function as chemical messengers. Among these molecules, IFNs are the most effective in elucidating antiviral immune responses [1]. Additionally, cytokines and chemokines also play important roles as chemoattractants controlling leukocytes trafficking. Innate immune response operates through the steps of recognition of the pathogen, signal transduction, and subsequent gene expression to produce the innate immune effector molecules.

The first step is to recognize a pathogen as a foreign object and differentiate it from self-components. When pathogens breach physical barriers such as the skin or oral mucosa, they are recognized by pattern recognition receptors (PRRs) expressed either in the cytoplasm or on cell membranes. PRRs sense and interact with the structurally conserved motifs of proteins and nucleic acids unique to invading pathogens known as pathogen-associated molecular patterns (PAMPs) [2]. The most widely studied PRRs, the toll-like receptors (TLRs), present either on the cell surface or in the endoplasmic compartments, are involved in recognizing microbial PAMPs. For example, TLR2 and TLR4 respond to specific viral glycoproteins; TLR9, TLR3/7, and TLR8 are involved in sensing viral nucleic acids as well as the unmethylated CpG sequence in viral DNA molecules [3]. In addition to TLRs, viral PAMPs are also detected by other PRRs including RIG-like receptors (RLRs), RIG-I, MDA5, C-type lectin receptor (CLR), and DC-SIGN. RIG-I and MDA5 recognize 5′ phosphorylated short and long dsRNA, respectively, whereas, DC-SIGN binds to viral envelop glycoproteins. Cytosolic receptors such as AIM2 and DAI are also identified as respondents of dsDNA [4]. The interaction of viral ligands with host receptors activates the downstream signaling events that in turn switch on specific transcription factors regulating the expression of genes responsible for innate and adaptive immunity interchange. For example, when TLRs bind to viral PAMPs, the intracellular part of TLR binds to MyD88 and activates mitogen activated protein kinase (MAPK) that leads to the activation of NF-*κ*B. Activation of NF-*κ*B promotes regulation of inflammatory cytokine genes and activates interferon regulatory factor (IRF) [5, 6]. IRF induces type I IFNs that function as antiviral and inflammatory agents [7]. Furthermore, IFN is also involved in maturation of DC and regulates the function of macrophages, NK cells, and T and B cells [8]. However, the efficacy of host response depends on a rapid and specific recognition response to invading pathogens. Upon infection, immune cells are activated to modulate their molecular networks to eliminate the pathogen.

## 2. Strategies of HIV-1 to Evade Innate Immune Response

To overcome these immune effector functions, viruses including retroviruses are evolved to counteract and subvert through various mechanisms [9, 10]. Retroviruses are a diverse family of enveloped RNA viruses that have the ability to evade immune defense system and establish long-term persistence in the infected hosts. HIV-1, one of the retroviruses, utilizes varying strategies compared to other viruses as the PAMPs exposed by the virus are not easily recognized by the PRRs, and hence the virus can escape from proinflammatory and antiviral responses [11]. One possible mechanism by which HIV-1 manages to avoid immune encounter of the host is that HIV-1 can modify its PAMPs by altering or hiding its nucleic acids in the viral capsid in order to mimic the cellular proteins [12]. The ability of genetic variability of HIV-1 is one of the major immune evasion strategies for the virus. The HIV-1 RNA genome can be mutated randomly which helps the virus to evade immune recognition by the host. Error prone viral reverse transcriptase lacking the proofreading activity is responsible for the high mutation rate in HIV-1 [13]. By avoiding the immune recognition, HIV-1 crosses various checkpoints in innate as well as adaptive immune defense machineries of the host. This review emphasizes on some of the mechanisms utilized by HIV-1 to escape host's innate antiviral responses.

## 3. Evasion of HIV-1 through Mucosal Barriers during Early Infection

HIV-1 enters primarily through the mucosal surfaces of genital or rectal tissues during sexual transmission. Mucosa presents the first line of physical defense against invading pathogens. HIV-1 evades this initial barrier by successfully crossing the mucosa to reach the susceptible host cells and establish the infection. One of the proposed mechanisms is that the virus crosses the mucosal barrier by transcytosis or by capturing dendrites of intraepithelial DCs [14]. Additionally, HIV-1 also utilizes the intercellular spaces to move through the epithelium and achieve contact with the underlying mucosal Langerhans cells and CD4^+^ T cells [14]. Exposure of HIV-1 or the viral protein gp120 disrupts tight junction proteins and the monolayer integrity of mucosal epithelium by the upregulating inflammatory cytokines that leads to the increased permeability of the virus particles [15]. These studies suggest that HIV-1 may induce defects at the mucosal epithelial barrier, which could activate mucosal T cells and increase the production of inflammatory cytokines [16–18]. A recent study demonstrated the association of *γδ* T cells as a major component of mucosal immune system with the early HIV-1 induced events [19]. Breakdown of mucosal barrier is considered as the most crucial event causing HIV-1-associated immune activation. 

Following mucosal breaching, HIV-1 establishes acute infection in immune cells present within the mucosa. It has been suggested that the productive HIV-1 infection starts from a single infectious virus particle [20, 21], and the resting CD4^+^ T cells are the first targets [21–23]. These infected cells disseminate with the help of proinflammatory cytokines such as IL-1, IL-8, IL-6, and GM-CSF to the lymphoid tissues throughout the body including the gut-associated lymphoid tissue (GALT) containing high numbers of CD4^+^ T cells where the virus replicates at a very high rate. A group of freshly infected CD4^+^ T cells is generated, thus inducing rapid spread of HIV-1. This results in a peak of viremia or a viral set point followed by induction of CD8^+^ T lymphocytes, and dramatic loss of CD4^+^ T cells. Finally the viral load is controlled and maintained at a steady level throughout the chronic phase of HIV-1 infection. Hence, evading the physical barrier posed by the mucosal tissues marks the success of the initial stages of viral infection and spread. 

## 4. Complement System 

After penetrating the initial mucosal barrier, complement system confers a major host defense mechanism contributing the restriction in viral replication by triggering the recruitment of inflammatory cells and also by rupturing plasma membranes of undesired cells. Complement system functions as inhibitor as well as beneficiary for HIV-1 infection and pathogenesis. Complement pathways lyse HIV-1 particles and the infected cells to neutralize IgG and IgM-bound viruses. However, HIV-1 overcomes the complement mediated inhibition of viral spread or pathogenesis by activating the classical pathway of complement system by binding C1q with envelope protein gp41 [24]. HIV-1 downregulates expression of host complement receptors that impair monocyte chemotactic responses to inflammatory stimuli (exposure of gp120 decreases C5a expression) [25]. Deposition of C3 and C5a facilitates HIV-1 interaction with complement receptor CR3 and CR4 containing cells including monocytes/macrophages and DCs. HIV-1 gp41 interacts with CR3 and this interaction enhances both viral entry and viral spread in the cells [26]. HIV-1 also binds to CR1 on erythrocytes and CR2 on B cells and exploits these cells to generate C3d-opsonized infectious HIV-1 reservoirs and to spread infection to the uninfected organs [27–29]. In addition to increasing viral infectivity, HIV-1 gp41 and other viral proteins also stimulate the synthesis of C3 in neurons and astrocytes [30]. This increased production of complements is another means of contributing HIV-1-induced neuropathogenesis. Also, gp41 and gp120 recruit factor H, which is responsible for protecting self-cells from complement mediated lysis to multiple binding sites [31, 32]; thus, in turn, it causes decreased complement dependent lysis of infected cells and virus *in vitro* [33]. Another complement regulatory factor CD59 present on HIV-1 envelope prevents complement mediated neutralization of antibody bound viruses [34]. Together, these studies indicate that HIV-1 has evolved to override the complement system induced innate antiviral response to increase viral spread.

## 5. HIV-1 Induced Changes in Cytokine and Chemokine Profiles

Cytokines and chemokines have the most influential role in HIV-1 pathogenesis, and the virus exploits the network throughout its life cycle. HIV-1 infection results in the activation of immune cells, altered functions of macrophages, NK cells, and DCs, and the activation of B cells [35–41]. Overactivation of immune cells leads to increased production of pro-inflammatory anti-inflammatory cytokines and chemokines including IFNs, tumor necrosis factor (TNF)-*α*, interleukin- (IL)-1, -2, -4, -8, -6, -10, -15, interferon gamma-induced protein (IP)-10, and monocyte chemotactic protein (MCP)-1 [42, 43]. These cytokines/chemokines either enhance or inhibit HIV-1 replication. With disease progression—a shift from stimulatory Th1 (IL-2, IFN) to inhibitory Th2 (IL-4, IL-10, IL-1, IL-6, IL-8, and TNF), cytokine production takes place [44]. Attachment of HIV-1 envelope protein, gp120, triggers production of CC chemokines CCL2, CCL3, CCL4, and CCL5 that are chemoattractants for DCs, macrophages, and lymphocytes. The expression of these CC chemokines is regulated by cytokines including IL-6, TNF-*α*, IL-1*β*, and IL-10, which are also regulated by HIV-1 infection [45]. HIV-1 utilizes different mechanisms to override host cytokine/chemokine networks; for example, HIV-1 transactivator of transcription (Tat) protein mimics *β*-chemokines and functions as chemoattractant of monocytes/macrophages that leads to increased activation and infection [46]. A previous study has shown which HIV-1 negative regulatory factor (Nef) increases the production and the stimulatory function of proinflammatory cytokines such as IL-1*β*, IL-12, IL-15, and TNF-*α* and chemokines such as macrophage inflammatory protein (MIP)-1*α*, -1*β* and IL-8 when it interacts with immature DCs [47, 48]. HIV-1 accessory protein viral protein R (Vpr) also alters the level of proinflammatory cytokines [48]. Hence, HIV-1 either by mimicking or by modulating certain cytokines exploits the cytokine network for its replication and survival. 

## 6. Interferons and Signaling Events

Among the cytokines, IFNs play the major role in virus infection and confer the first defense. Type I IFNs (include IFN-*α*, -*β*, -*ε*, -*κ*, and -*ω*), the innate cytokines produced by innate immune stimuli, have multiple properties including induction of immune activation, enhanced antigen presentation, and antiviral activity [49]. IFNs are mainly produced by IFN regulatory factors (IRFs) especially IRF3 and IRF7 [50]. They bind to IFN receptor IFNAR1/IFNAR2 at the cell surface and form STAT1/STAT2 dimer, which translocates into the nucleus and triggers downstream signaling pathways that result in activation of interferon-stimulated genes (ISGs). Cellular factors, ISGs, generated from type I and type II IFN, induce signaling events that inhibit HIV-1 replication [51, 52]. Recognition of PAMPs stimulates activation of IFN-induced signaling pathways triggering activation of transcriptions factors including NF-*κ*B, activator protein (AP)-1 and IRF3, which in turn leads to production of proinflammatory cytokines and chemokines and antiviral type I IFN. The innate immune response largely depends on virus-induced signaling pathways to induce IFN response. A recent study indicated the role of tripartite motif containing 22 (TRIM22) induced by IFN-*β* as an inhibitor of HIV-1 replication [53].

Since IFN has the most potent role in preventing viral replication and eliminating infection, HIV-1 blocks and/or minimizes the IFN production as well as the downstream signaling pathways to prevent immune responses. However, the mechanism(s) by which HIV-1 can manage to escape IFN induced anti-HIV-1 activity is not fully understood. It was documented that HIV-1 gp120 blocks cytokines including IFN and the cytolytic activity of NK cells by interfering with TLR9 activation in pDCs [54]. In a SIV model of central nervous system, it has been shown that SIV infected astrocytes produce monocyte chemotactic protein (MCP)-1 or CCL2 that binds to the CCR2 receptors on macrophages resulting in suppression of specific ISGs such as TRAIL [55]. In macrophages, HIV-1 exploits another cellular factor, suppressor of cytokine signaling (SOCS) 3 to facilitate its replication. SOCS3 inhibits IFN-*β* signaling in macrophages, and hence it prevents antiviral gene expression that helps HIV-1 replication [56]. Studies also indicated that cellular 3′ repair exonuclease 1 (TREX1) helps the virus to hide from immune activation [57]. The exonuclease activity of TREX1 degrades excess ssDNA of HIV-1 and thus prevents activation of IFN by this excess HIV-1 DNA. Mutation or dysfunction of TREX1 results in accumulation of HIV-1 DNA and triggers DNA sensors. This activates STING, TBK1, and IRF3 transcription factors. Activated IRF3 translocates into nucleus and enhances IFN response by activating the respective promoters in different cell types [57–59]. 

The release of IFNs leads to the induction of ISG through JAK/STAT pathways. ISGs induce antiviral response. One of the first ISG linked to antiviral response is the IFN-induced protein kinase R (PKR). It plays an important role, when it binds to dsRNA of viral replication product, forms a dimer by autophosphorylation, and blocks viral replication by inhibiting translation of alpha subunit of elongation initiation factor 2 (eIF2*α*) in infected cells. HIV-1 infection does not activate PKR. Virus replicates in cells where a high level of TAR binding protein (TRBP) is present. TRBP functions as a strong inhibitor of PKR and eIF2*α* [60, 61]. TRBP also binds to and serves as a cofactor of Dicer, which is required for biosynthesis of microRNA (miRNA) or RNA interference (RNAi) [62], inhibitors of viral replication. HIV-1 overrides the PKR mediated innate response via two different mechanisms. First, HIV-1 Tat RNA binds to TRBP and inhibits PKR activation; also TRBP becomes unavailable for Dicer binding [63]. Thus HIV-1 exploits TRBP as a link between IFN and RNAi mediated antiviral response. Second, HIV-1 Tat also functions as a substrate homologue of eIF2*α* and competes for PKR mediated phosphorylation. PKR phosphorylates HIV-1 Tat, and the phosphorylation of Tat is necessary for HIV-1 LTR transactivation [64]. This prevents phosphorylation of eIF2*α* and enhances viral replication [65]. Another protein adenosine deaminase acting on RNA (ADAR1) is also reported to inhibit PKR activation [60]. Together, these reports support that HIV-1 proteins play a major role in combating IFN responses, thus blocking innate antiviral activity.

## 7. Plasmacytoid Dendritic Cells (pDCs) Function as Major Source of IFN

After HIV-1 entry into the system, the immediate IFN response is primarily from pDCs. pDCs play the central role in innate immune response against viral pathogens through the secretion of enormous amount of IFN. It expresses TLR7 and TLR9 in addition to CD4, CCR5, and CXCR4 receptors. TLR7 and TLR9 on pDCs bind to ssRNA and unmethylated CpG DNA motif, respectively, and signal through MyD88 that in turn leads to the activation of IRF7 for IFN production. Compared to other blood cells, these cells produce several times more type I IFNs that function as immunostimulatory cytokine to drive mDC maturation [66]. HIV-1 inhibits the innate immune response of pDCs by reducing pDC cell counts in peripheral blood. HIV-1 infected individuals are reported to have lower levels of pDCs compared to uninfected individuals [39]. HIV-1 gp120 exposure suppresses pDC activation and production of proinflammatory cytokines mediated through TLR9 [54]. Other studies have demonstrated that HIV-1 blocks pDC function by suppressing TLR7 and TLR8 [67] and also by inhibiting IFN-*α* [68]. However, before undergoing HIV-1-induced cell death, pDCs upregulate CCR7 production, accumulate in lymph node, and produce high level of IFN [69]. High plasma level of IFN has been observed during acute HIV-1 infection and also during the late stage of the HIV-1 disease. It has also been reported that IFN enhances disease progression to AIDS [70]. HIV-1 induced production of IFN by pDCs leads to the expression of TNF-related apoptosis inducing ligand (TRAIL) [71], which is involved in triggering apoptosis of uninfected CD4^+^ T cells [72]. Through TLR7, TRAIL turns pDCs into IFN-producing killer pDCs (IKpDCs) that produce high level of TNF-*α* [73]. It has also been shown that in addition to IFNs activation of pDCs, HIV-1 also stimulates production of inflammatory cytokines/chemokines including TNF-*α*, IL-6, CXCL10, CCL4, and CCL5 [74, 75].

## 8. Functional Dysregulation of Natural Killer (NK) Cells by HIV-1

The immunomodulatory and cytotoxic activities of NK cells are impaired in HIV-1 infected individuals, diminishing the innate and detrimentally affecting the adaptive immune response [76–79]. Analysis of frequency, phenotypes, and function of peripheral blood CD3-CD56+NK subsets in HIV-1+ individuals revealed significantly reduced numbers of total NK cells and a striking shift in NK cell subsets. More specifically, CD56^dim⁡^ populations of HIV-1 infected subjects were markedly diminished (and exhibited functional abnormalities) when compared to IFN-*γ* producing CD56^bright^ NK cell fractions from the same individuals [79]. HIV-1 infection is characterized by a dramatic increase in inhibitory receptors and loss of activating receptors, particularly, NKp30 on NK cells, resulting in loss of NK cell activity and defective crosstalk with DC [80, 81]. Involvement of HIV-1 in the impairment of NK cell function *in vivo* has also been bolstered indicating an association between NK cell ligand HLA-B Bw4-801 and its receptor; the killer immunoglobulin-like receptor (KIR) 3DS1; expression of HLA/KIR subtypes can be linked to efficient NK cell mediated inhibition of HIV-1 replication and killing of infected targets [82, 83]. 

The observed NK cell dysregulation is attributed to HIV-1 viral proteins [84, 85]. HIV-1 Nef functions as a potential regulator of NK cell cytotoxicity due to its involvement in MHC class I downregulation on CD4^+^ cells [86]. The selective downregulation by Nef of HLA A and HLA B but not HLA C or HLA E molecules in infected target cells inhibits NK cell cytotoxicity which confers an additional evasion strategy targeting the antiviral activities of NK cells [87–89]. HIV-1 Tat inhibits LFA-I-mediated Ca^2+^ influx through the binding of L-type Ca^2+^ channel and thereby impairs NK cell cytotoxicity [90, 91]. A linear motif of HIV-1 gp41 induces expression of NKp44L on CD4^+^ T cells and results in their depletion by selective lysis by NK44^+^ NK cells [37]. Azzoni et al. showed that virally suppressed children had normal levels of circulating pDCs, mDC, and total NK cells, yet they had sustained depletion of mature NK cell subsets along with diminished IFN-*α* production by pDC, which is required to enhance NK cell cytolytic responses [92]. HIV-1 treated target cells exhibit selective upregulation of NKG2D receptor on NK cells, and this upregulation is contact dependent and reversible [93]. Expression of these molecules is directly correlated with efficient killing of infected targets as well as controlling viremia, suggesting that innate immune cells play a critical role in immune control.

## 9. Antiviral Host Restriction Factors

In addition to other innate antiviral factors and innate immune cells, host restriction factors have also been shown to restrict HIV-1 infection in multiple ways. For instance, apolipoprotein B editing catalytic polypeptide (APOBEC3) is a member of cytidine deaminase family that has specific anti-HIV-1 property. APOBEC3G incorporates into HIV-1 through interacting with Gag and restricts HIV-1 infection [94, 95]. It mutates cytidine to uridine in negative sense single stranded DNA by deamination resulting in guanosine to adenosine hypermutation in positive sense viral cDNA and thereby makes it vulnerable to nuclease degradation [96, 97]. Current studies show that APOBEC3G functions in deaminase independent manner [98]. However, HIV-1 viral infectivity factor (Vif) protein inhibits the packaging of APOBEC3G in virus producer cells by functioning as an adaptor molecule that links a cullin-5-based E3 ubiquitin ligase complex with APOBEC3G leading to polyubiquitination and targeting APOBEC3G and APOBEC3F to proteosomal degradation [99–101].

Another restriction factor implicated in early steps of HIV-1 replication is tripartite motif-containing 5 (TRIM5). TRIM5 functions as E3 ubiquitin ligase and recognizes the motifs in viral capsid in the cytoplasm of an infected cell. TRIM5 prevents reverse transcription and transports viral genome to the nucleus [102]. Although the exact mechanism of TRIM5 inhibition is unknown, it has been suggested that TRIM5 promotes innate immune signaling, which is enhanced by interaction with viral capsid lattice [103]. TRIM5 binds to the viral capsid and activates the protein's ubiquitin ligase activity resulting in the synthesis of ubiquitin chains. These chains stimulate TAK1 (also known as MAP3 K7) phosphorylation followed by expression of NF-*κ*B-dependent genes. In primates, TRIM5 expression regulates expression of genes by AP-1 and NF-*κ*B activation [103, 104]. 

Tetherin is another intrinsic restriction factor involved in innate antiviral response. Tetherin/BST2, a glycosylated type II transmembrane protein, prevents budding of newly formed virus particles from plasma membrane of HIV-1 infected cells. Tetherin restricts HIV-1 transmission from one cell to another [105]. Its expression is upregulated by type I IFN. HIV-1 viral protein unique (Vpu) induces detachment of virus particles from cell membrane [106, 107]. Vpu interacts directly or indirectly with tetherin and reduces its expression at the surface. Viruses that are defective in Vpu expression remain tethered to the cell surface [11]. These tethered virus particles are subjected to endocytosis and degraded in lysosomes [12]. Recent studies indicated that the Vpu-like activity has been reported for other viral proteins including Env and Nef [13, 108, 109]. 

## 10. Other Regulatory Factors

Studies have shown that HIV-1 infection as well as innate immune response to viral infection could be controlled by host miRNAs [110]. miRNAs are short ~20–22 nucleotide long small noncoding RNA molecules expressed in most organisms [111]. These miRNAs control gene expression by binding to the 3′ UTR region of the target mRNAs. They can regulate the expression of both cellular and viral genes. For example, miR-26a, -34a, 145, and let-7b have been reported to regulate IFN-*β* in human and macaque cells [112]. Distinct differences in miRNA profiles have been reported in PBMCs infected with HIV-1 compared to control [113]. HIV-1 replication involves downregulation of specific cellular miRNAs (e.g., miR-29), which plays a significant role in controlling HIV-1 life cycle. Hsa-miR-29 binds to the conserved sequence of Nef 3′-LTR, transports HIV-1 mRNA to P-bodies and inhibits translation of viral mRNA [114]. Other miRNAs including miR-150, 223, 198, and 382 have been shown to be downregulated in HIV-1 infected macrophages and are known as anti-HIV-1 miRNAs, as they exert negative effect on the viral replication [115]. Treatment of macrophages with IFN-*α* and -*β* increases the expression of these miRNAs confirming their role in HIV-1 replication [56]. Alternatively, HIV-1 has also evolved viral miRNA to counterattack the host machineries. HIV-1 Tat interacts with Dicer, inhibits its activity, and suppresses the miRNA synthesis [116]. HIV-1 also blocks RNA silencing pathways by TRBP [62, 63]. Some viral miRNAs such as TAR miRNA, Nef miRNA (miR-N367), and miR-H1 are expressed, and regulate viral as well as host gene expression to facilitate virus replication and establishment of latency [117]. 

## 11. Conclusion

The interaction between host and HIV-1 is complex. The host system utilizes its machinery to defend HIV-1 infection, whereas the virus uses the host's tools as means of its own propagation. Starting at the transmission site (mucosal barrier) to immune activation site (lymph node), HIV-1 utilizes very unique strategies such as modification of its PAMPs, rapid mutation in its RNA to escape immune recognition, downregulation of complement receptors, increased secretion of inflammatory factors, and downregulation of NK cell function to overcome the innate immune response. The role of HIV-1 viral proteins to overcome host innate immunity is specified in Table 1. Current research focuses on understanding how HIV-1 overrides the innate immune system at multiple levels. The understanding of how HIV-1 prevails the host innate immune defense will increase our knowledge that could improve developing therapeutic approach to resist HIV-1 infection and spreading.

## Figures and Tables

**Table 1:** Role of HIV-1 viral proteins in innate immune evasion.

HIV-1 accessory proteins	Function in innate immune evasion
gp41	(i) Activates classical pathway of complement system (ii) Enhances viral entry and spread through interaction with complement receptor CR3 (iii) Stimulates synthesis of C3 in neurons and astrocytes (iv) Recruits factor H responsible for protecting from complement dependent lysis (v) Induces expression of NKp44L on CD^4+^ T cells and results in their depletion
gp120	(i) Recruits factor H (ii) Induces production of inflammatory cytokines and the CC chemokines (iii) Suppresses pDC activation and produces IFN and other cytokines (iv) Inhibits cytolytic activity of NK cells by interfering with TLR9
Tat	(i) Substrate homologue for eIF2*α* and competes for PKR mediated phosphorylation (ii) Mimics *β*-chemokines and functions as chemoattractant for monocytes and macrophages (iii) Inhibits LFA-I mediated Ca^2+^ influx and impairs NK cell cytotoxicity (iv) Interacts with Dicer and inhibits its activity, suppresses miRNA synthesis
Nef	(i) Increases expression of proinflammatory cytokines (ii) Downregulates HLA-A, HLA-B on target cells and inhibits NK cell cytotoxicity (iii) Helps in budding of HIV-1 (iv) Produces viral miRNA in HIV-1 persistently infected cells
Vpr	(i) Alters the levels of proinflammatory cytokines (ii) Upregulates NKG2D receptor on NK cells
Vpu	(i) Causes detachment of viral particle from cell membrane through interaction with tetherin
Vif	(i) Inhibits the packaging of APOBEC3G in virus producer cells causes proteosomal degradation of APOBEC3G
